# Design of a Digital Comic Creator (It’s Me) to Facilitate Social Skills Training for Children With Autism Spectrum Disorder: Design Research Approach

**DOI:** 10.2196/17260

**Published:** 2020-07-10

**Authors:** Gijs Terlouw, Job TB van 't Veer, Jelle T Prins, Derek A Kuipers, Jean-Pierre E N Pierie

**Affiliations:** 1 Academy of Healthcare Digital Health Care Research Group NHL Stenden University of Applied Sciences Leeuwarden Netherlands; 2 Medical Faculty Lifelong Learning, Education & Assessment Research Network University Medical Center Groningen University of Groningen Groningen Netherlands; 3 Digital Health Care Research Group NHL Stenden University of Applied Sciences Leeuwarden Netherlands; 4 Serious Gaming Research Group NHL Stenden University of Applied Sciences Leeuwarden Netherlands; 5 Surgery Department Medical Center Leeuwarden Leeuwarden Netherlands; 6 Post Graduate School of Medicine University Medical Center Groningen University of Groningen Groningen Netherlands

**Keywords:** autism, serious media, boundary object, comic, design research

## Abstract

**Background:**

Children with autism spectrum disorder (ASD) often face difficulties in social situations and are often lagging in terms of social skills. Many interventions designed for children with ASD emphasize improving social skills. Although many interventions demonstrate that targeted social skills can be improved in clinical settings, developed social skills are not necessarily applied in children's daily lives at school, sometimes because classmates continue to show negative bias toward children with ASD. Children with ASD do not blame the difficult social situations they encounter on their lack of social skills; their main goal is to be accepted by peers.

**Objective:**

This study aims to design a comic creator—*It's me*—that would create comics to serve as transformational boundary objects to facilitate and enact a horizontal interaction structure between high-functioning children with ASD and their peers, aiming to increase mutual understanding between children at school.

**Methods:**

This research project and this study are structured around the Design Research Framework in order to develop the comic through an iterative-incremental process. Three test sessions, which included 13, 6, and 47 children, respectively, were initiated where the focus shifted in time from usability during the first two tests to the initial assessment of acceptance and feasibility in the third session. A stakeholder review, which included six experts, took place after the second test session.

**Results:**

A digital comic creator, *It's me*, was produced within this study. Children can create their own personal comic by filling in a digital questionnaire. Based on concepts of peer support, psychoeducation, and horizontal interaction, *It's me* has a rigorous base of underlying concepts that have been translated into design. Based on the first test sessions, the comic has shown its potential to initiate personal conversations between children. Teachers are convinced that *It's me* can be of added value in their classrooms.

**Conclusions:**

*It's me* aims to initiate more in-depth conversations between peers, which should lead to more mutual understanding and better relationships between children with ASD and their peers. The first test sessions showed that *It's me* has the potential to enact horizontal interaction and greater understanding among peers. *It's me* was designed as a boundary object, aiming to connect the objectives of different stakeholders, and to trigger reflection and transformation learning mechanisms. The applied design research approach might be of added value in the acceptance and adoption of the intervention because children, professionals, and teachers see added value in the tool, each from their own perspectives.

## Introduction

### Background

Children with autism spectrum disorder (ASD) often face difficulties in social situations and are often lagging in terms of social skills [1,2]. In social situations, children with ASD find it difficult to interpret verbal and nonverbal behavior and face difficulties in maintaining conversations and understanding the intentions of others [3-5]. Children with ASD show a lack of intuitive judgments within social contexts [3,6], often avoid eye contact [7-9], and do not spontaneously interact with other people [3,4]. Children with ASD have a higher risk of developing depressive or anxious feelings [10]. Many interventions designed for children with ASD emphasize improving social skills [11], with the aim, among other things, of being more in tune with peers. However, in social contexts, such as school, children with ASD are more likely to be victims of peer harassment and are more likely to be excluded by peers [12-17]. Although many interventions demonstrate that targeted social skills can be improved in clinical settings, developed social skills are not necessarily applied in children's daily lives at school [11,18]. Even when participants do improve social skills, sometimes classmates continue to show negative bias toward children with ASD [19]. In our earlier research, we reported that high-functioning children with ASD from 10 to 12 years of age do not blame the difficult situations they encounter at school on their lack of social skills [20]. Their main goal is to be accepted by peers and be a part of the group. Children with ASD do not necessarily see the link between improving their social skills and pursuing that goal. Because the classroom is the primary setting in which peer rejection is determined [19], interventions that incorporate the peer-group context can be of added value alongside traditional social skills training for children with ASD.

In autism research, there is a growing interest in the development of digital interventions [21]. Developed interventions range from simulation-based interventions for practicing social skills [22], use of smart glasses for coaching users in social communication [23], interventions using virtual reality [24-26], interventions based on a gamification approach [27-29], social training interventions using social media [30], and interventions aiming to train users with skills within a serious game [31,32]. Most of those interventions focus on the child with ASD and aim to develop a set of specific skills. Those interventions are often based on a traditional skills-based approach, just like most of their analog counterparts. Within a skills-based approach in social skills training, basic social skills, such as greeting, turn-taking, emotion recognition, and asking questions, are essential developmental goals [33,34]. Those interventions frequently take place outside the natural environment of the child, which reduces the opportunity to practice the learned social skills in natural settings [35]. School is the pre-eminent place where the skills learned should be put into practice.

In a skills-based approach, the professionals, but sometimes also the peers, are often positioned in the role of an expert who models and prompts desired social behaviors [34,36]. In doing so, this approach is not focused on facilitating a horizontal interaction structure between peers, because peers have a different status than the children with ASD. Interventions that create a vertical interaction structure can be useful to train specific skills but can limit the establishment of friendships and peer acceptance. Within a horizontal interaction structure, children have equal status within the interaction. Finke [37] specifically describes the limitations of a skills-based approach for the development of peer acceptance and friendship, where equivalence should be the starting point. Finke [37] notes the need to design instructional opportunities that “expose the knowledge, skills, and abilities of the individual with ASD” to facilitate “a horizontal interaction structure” across peers. Activities that include interactions that require members to have equal status, as well as ones that trigger members to communicate individuating information about themselves that disconfirms prevailing stereotypes, can increase social acceptance among group members [38]. For the design of a new intervention to enhance peer acceptance, these concepts offer a good starting point.

### Boundary Objects

Over the past decades, there has been a growing interest in boundaries, boundary crossing, and boundary objects [39-41]. A boundary can be seen as a sociocultural difference between different groups or sites. Boundaries suggest continuity and discontinuity between sociocultural sites at the same time. In the example of social skills training for children with ASD, the children with ASD and professionals who facilitate social skills training are connected and have a shared concern. Both parties have an interest in making the children function better in social situations, but the children have different goals to pursue than do the professionals. Professionals mainly focus on improving social skills, while children want to establish equal relationships with their peers. In addition to the boundaries that exist between the child with ASD and the professional, there are also boundaries within the contexts in which the child functions. At school, for example, there is a boundary between children with and without ASD, because they often do not understand each other properly in social interactions.

**Figure 1:**
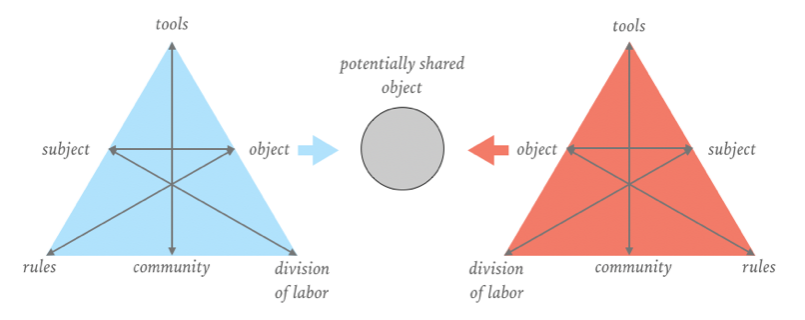
Boundary objects.

To bridge boundaries, boundary objects (see Figure 1) [41] can fulfill an important function. Many authors [40-43] have recognized the importance of boundary objects to support social interaction, to connect different sites, and to enable a shared understanding among different groups, sites, persons, or stakeholders. Boundary objects are flexible in adapting to the local needs and constraints of different parties. The acknowledgment and discussion of those differences are key to enabling a shared understanding among different sites or groups [44].

Akkerman and Bakker [40] describe four learning mechanisms that can take place at boundaries: identification, coordination, reflection, and transformation. Typical in identification processes is that the boundaries between sites are encountered and reconstructed, enhancing renewed sensemaking without necessarily overcoming discontinuities. The coordination mechanism establishes effortless movement and constructive alignment between different sites, only as far as necessary. The reflection mechanism enacts an expanded set of perspectives and new construction of identities. The transformation mechanism can entail or develop new, in-between practices and sites. Most of the current social skills interventions seem to focus on strengthening the children's coordination learning mechanisms, where the child learns skills as tools to interact as smoothly as possible with other sociocultural systems. The reflection and transformation mechanisms are seldom used, while it is precisely these mechanisms that could lead to a rebalancing of relationships between children with ASD and their peers, whereby individuals understand each other's perspectives and can construct a good way of interacting with each other. From the reflection and transformation mechanisms, the child with ASD would not only have to adapt to the systems around him or her, but the systems around him or her would also adapt to the child. However, a solid boundary object will be needed to enact these mechanisms.

### Comics as Boundary Objects

Comics are a narrative medium that integrates text and visual imagery [45]. Comics can trigger specific affective processes of, and responses by, readers [46,47], which makes them suitable to tell complex and emotionally rich stories [48] without requiring an individual to read long or complicated texts. Good comics should be able to enact narrative transportation. Narrative transportation occurs whenever a reader experiences a feeling of empathy for the characters and has a vivid imagination of the story plot [49,50]. When carefully composed, comics can, therefore, be a novel and creative way to learn and teach about difficult themes, such as illness or disabilities [48]. In health care, comics are already used to promote public awareness and enhance patient care for various problems, including cancer [47,51,52], HIV [53], diabetes [54], and mental illness [55].

This study aims to design a digital comic creator—*It’s me—*that would create comics to serve as transformational boundary objects, which would facilitate and enact a horizontal interaction structure between high-functioning children with ASD and their peers. This creation should serve as a support tool to initiate more in-depth conversations between children about what they are good at as well as about areas where they perceive more difficulties, whether or not this is because of their disability. This tool aims to increase mutual understanding between children with ASD and their peers to create a safe environment at school. This intervention intends to be complementary to existing interventions and is based on a different perspective and approach. This paper describes the design research process of the tool and the first field tests that were used to investigate usability, acceptance, and feasibility.

## Methods

### Study Design

**Figure 2:**
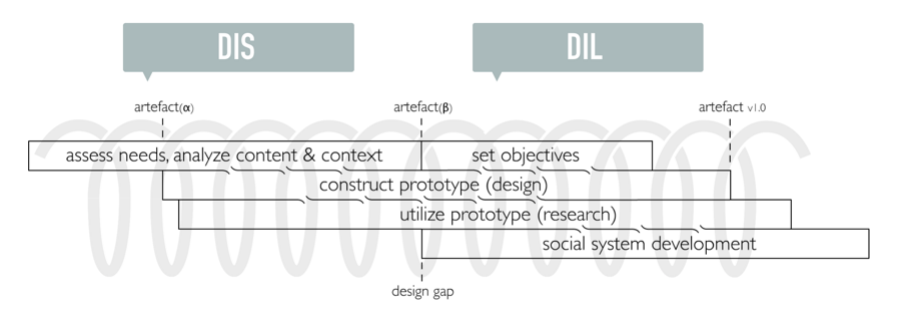
The Design Research Framework. DIL: design-in-the-large; DIS: design-in-the-small.

This research project and this study are structured around the Design Research Framework (see Figure 2) [56,57]. This framework facilitates the development of serious media interventions through an iterative-incremental process. The focus of these iterations shifts during the process, along with nonlinear design steps [58]. After the phase where the focus was on assessing needs, analyzing content, and context [20], this study focuses more on the construction and utilization of prototypes.

During the design process, the social system is frequently exposed to various design methods and prototypes. Those methods and prototypes are regarded as boundary objects [42], which help to map out the perspective of different stakeholders during the process. At the same time, they have an influence on the social system of stakeholders from a dialogical learning perspective. In our earlier research [20], we focused on the identification and coordination mechanisms. During this study, where the prototypes are becoming increasingly more mature, the focus in the social system will shift to the learning mechanisms of reflection and transformation. The ultimate goal of the transformation mechanism in this project is that the developed intervention will be able to disrupt existing cultural patterns and allow better interaction between high-functioning children with ASD and their peers.

In this study, several prototypes of *It’s me* were tested with children and other important stakeholders. Three test sessions were initiated, where the focus shifted in time from usability in the first two tests to the initial assessment of *early indicators of success* [59], acceptance, and perceived usefulness in the third test. Between the second and third test sessions, a stakeholder review took place.

### Test Session 1: Usability Tests

For the first test session, a mixed group of 13 children was selected; children were 10-12 years of age. A collaborating, primary special education school recruited the children. In the Netherlands, primary special education schools have the same core objectives and curriculum as regular primary schools. Yet, primary special education schools offer extra help for children with additional needs. The groups in primary special education schools are smaller, and there are more teachers and experts available to assist the children. Out of the 13 recruited children, 7 (54%) were diagnosed with ASD and 6 (46%) needed extra assistance for other causes. The children and their parents gave informed consent. All retrieved data have been processed anonymously.

**Figure 3:**
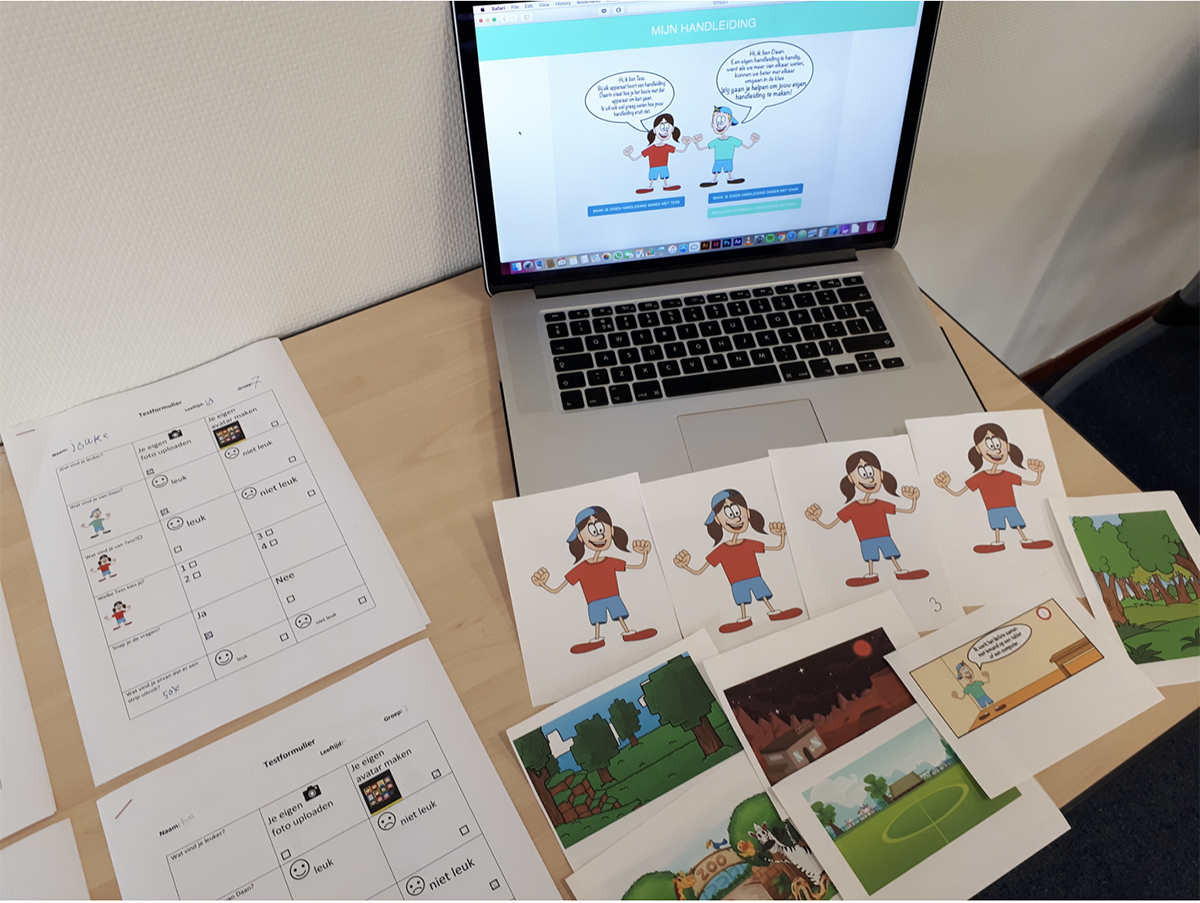
Worksheets, cards, and website prototype for test session 1.

**Figure 4:**
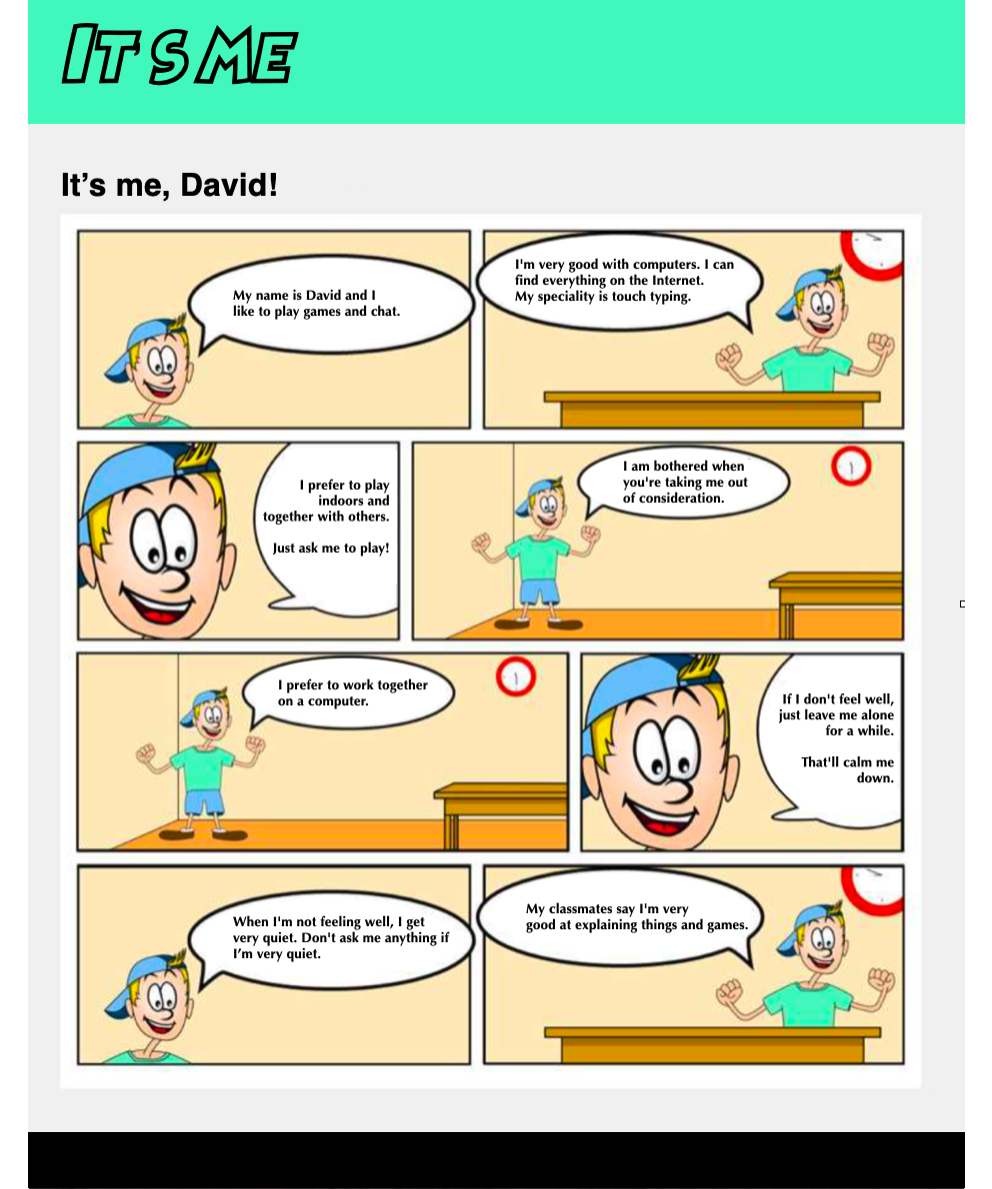
Comic strip prototype for test session 1.

For the first test session, we developed a prototype (see Figures 3 and 4). In this prototype, the children could fill in a questionnaire. Based on the answers they gave in the questionnaire, a personal comic was generated. The items consisted of questions and prompts and were composed by the authors together with six experts. Three of those experts work as social skills trainers in child psychiatry; they are familiar with social skills training in a clinical setting. The other three experts work at primary special education schools and are familiar with the target group and the challenges in social interactions that these children face at school. The questionnaire contained items about what the children are good at and where they perceive more difficulty. The items from the questionnaire can be found in Textbox 1. The main goal of this first test was to evaluate the prototype regarding usability, to check whether the children understood the items, and to receive feedback from the children on the look and feel of the comic. The children were also observed during the session. After the session, we interviewed the children about their experiences with the tool.

**Items from the questionnaire.**

1. What’s your name?
2. How old are you?
3. My hobbies are...
4. My favorite subject at school is...
5. I am good at...
6. Nobody knows that...
7. I prefer to play together / I prefer to play alone.
8. I prefer to play outside / I prefer to play inside.
9. What is the best way for you to be approached to play together?
10. I’m not very good with...
11. I can't stand it as well when...
12. In class, I don't like it when...
13. I prefer to work alone / I prefer to work together.
14. If I have to work together, it's best if...
15. What helps you get through a day at school?
16. If I don't feel good, you can tell by the way...
17. The best way to help me is by...
18. Peer: What's nice about...
19. Peer: What does your classmate do very well?

### Test Session 2: Cocreative Refining Session

**Figure 5:**
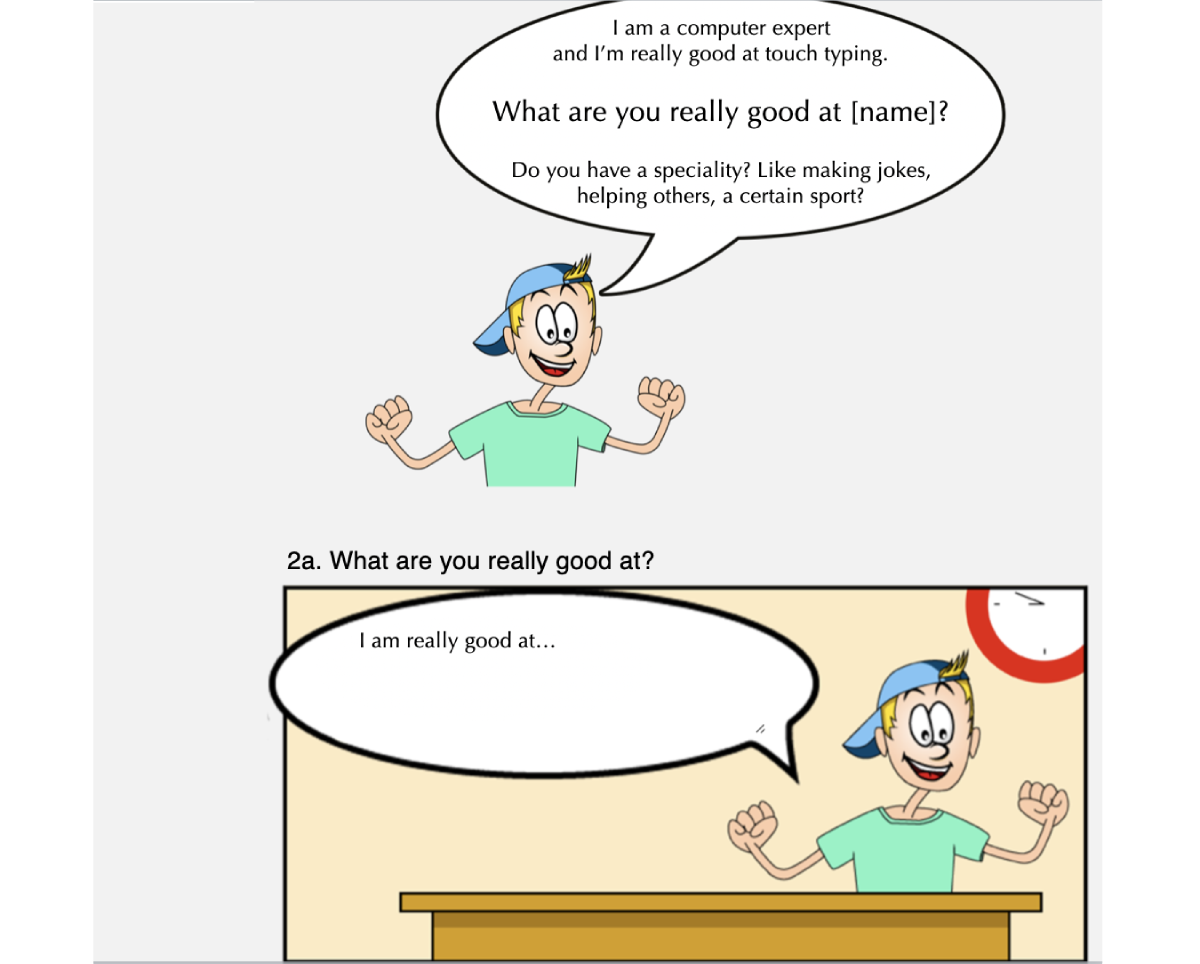
Example of a question with sample answers.

The second test session took place at the same primary special education school as did the first session. The school recruited 6 different children to participate; 3 (50%) of them were diagnosed with ASD and 3 (50%) of them were children who needed extra assistance for other causes. All the participating children were between 10 and 12 years old. For this second test session, modifications were made to the prototype. The main difference was the presentation of the items, where we added sample answers for clarification (see Figure 5). The children and their parents gave informed consent. All retrieved data have been processed anonymously.

One aim of the second session was to check whether the adjustments in the presentation of the items helped the children to better understand them. Another aim of the second session was to get feedback and input on the appearance of the comic. Through a creative working method, children made their own sketches and drawings to elaborate on the existing comic artwork, then they explained their drawings and paintings. The discussion about the elaborations the children made aimed to gather insights about what the children thought was important in the appearance of the comic.

### Expert Review

To ensure that the generated comics would provide enough material for in-depth conversations between peers afterward, six social skills training experts who work in child psychiatry were consulted to give their input; these were different experts than those in test session 1. During the session, the entire prototype, from the questionnaire to the generated comic strip, were reviewed step by step in a walk-through session. The experts were invited to share their initial reactions and findings during each step of the process. After the walk-through, the experts gave input regarding the content of the conversation that should take place between children based on the comic. The experts gave informed consent. All retrieved data have been processed anonymously.

### Test Session 3: Test and Evaluate

The third test aimed to investigate whether the designed intervention had enough potential for the intended purpose, whether children and other stakeholders accepted the tool, and whether the tool seemed to have added value for children and their teachers. In this phase of the design process, research was aimed at improving the design and at locating design constraints necessary to use the tool in a meaningful way. In the evaluation, it was decided not to make a distinction between children diagnosed with ASD and children not diagnosed with ASD. Both groups of children are considered equally important in this phase for examining acceptance and perceived usefulness, since the tool is intended to be used by both groups at the same time.

For the third test session, a mixed group of 47 children aged 10-12 years old participated; 19 of those children (40%) were diagnosed with ASD. A collaborating, primary special education school recruited the children; this was a different school than the one in the previous sessions. Four classes participated in this session. During the session, the full prototype was tested by the children. For the test, children were taken out of the classroom in groups of 4. This way of testing allowed regular schoolwork to continue as much as possible. During the test session, the groups were supervised and guided by two research assistants.

During the test, participating children first completed the questionnaire (see Textbox 1) individually on a tablet device. The children were observed by both research assistants while answering the questions. The research assistants scored the behavior of the children independently at two preset moments: after precisely 1 minute and after precisely 5 minutes. The observed behavior of the children was scored on verbal and nonverbal behavior regarding how they reacted to the questionnaire. The scoring categories were as follows: enthusiastic, disinterested, angry, insecure, and distracted. After 10 and 15 minutes, the children were scored on their social behavior. The scoring categories were as follows: helpful, approaching a peer, watching a peer, and making fun of someone. To increase the predetermined degree of reliability, the research assistants practiced the observing method by observing and scoring children during a regular school assignment, followed by exchanging and discussing the results. The research assistants repeated this several times until there was a high degree of consensus.

When all the children finished the questionnaire the comics were printed, and the children discussed their personal generated comics in groups of 4. During this phase, one research assistant focused on keeping the conversation going; the other one observed the children and scored their communicative behavior. The results were checked and discussed with the other research assistant afterward.

**Figure 6:**
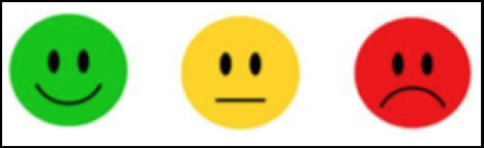
Scorecard used for answering closed questions during test session 3. The green smiley on the left indicates “yes,” the yellow smiley in the middle indicates “neutral,” and the red smiley on the right indicates “no”

At the end of the session, all participants were invited to partake in an evaluation session. A questionnaire was used to ask the children about their experiences. Three closed questions and one open question were asked about the use of the tool itself. Five closed and three open questions were asked about whether the children had learned something from each other and, as a result, had developed more understanding for each other. Closed questions were answered using smileys (see Figure 6). The scores are reported in the Results section.

After the test sessions with the children, their five teachers were interviewed in a semistructured interview. The teachers were questioned on three topics: (1) How do they evaluate the tool regarding its usefulness on face validity? (2) What do they notice about the children after using the tool? and (3) Would they like to use the tool in their professional practice in the future? The data from these interviews were analyzed using a general inductive approach [60].

The children, their parents, and the teachers gave informed consent. All retrieved data have been processed anonymously.

## Results

### Test Sessions 1 and 2

During the first two test sessions, the emphasis was on usability. The test sessions showed that the system has good overall usability. Children could easily use the tool and did not run into any problems when using the tool. However, it was noticeable that children with ASD interpreted some of the questions differently in the first version of the prototype. They often asked for help to clarify the questions or to get some sample answers. For this reason, sample answers were added to the prototype for the second test session. During the second test session, children needed less help.

During the first user tests, some early indicators of the intended purpose of the tool became visible. Children helped each other during the session, talked about their comics, and asked each other about their comics. The conversations quickly transcended the superficial, in which children actively gave each other feedback and exchanged how they interpreted certain behaviors by the children. For example, a peer pointed out that one of the children could not hide her frustration as well as she thought: “I can see it right away when you're frustrated.” The girl replied, “Really? I didn't think anybody saw that.” In another conversation, one of the children actively asked for help: “Especially when I am scowling; I often need some help.” His peer replied, “We thought we should leave you alone when you looked that way.” Those conversations arose quite naturally. It turned out, however, that children were hesitant when asked if they would like to share their comic strips with other classmates. An essential condition for the children should be that everyone will share their stories.

The main feedback from the children during the first two test sessions focused on the look and feel of the comic, which mainly manifested itself during the creative session in which children drew on the generated comics. According to the children, the characters were very simple, and the backgrounds were experienced as quite dull. The children also had a minimal choice in shaping their character; they could only choose between a boy or a girl. From the computer games they play and the cartoons they watch, children turned out to be accustomed to high-quality illustrations and many possibilities in personalizing their characters.

### Expert Review

The experts gave the prototype an overall positive review and had only a few concerns. Some concerns were about the way the questions and the sample answers were phrased. Based on the feedback from the experts, adjustments were made to the questions and sample answers. The experts also had some comments about the meaning of the visual aspect. One expert mentioned the following:

Children with ASD can sometimes pay a lot of attention to detail. If the cartoon character says he likes to play outside, but is inside in the illustration, a child with ASD can already disengage. Also, in the end, the cartoon character must be able to look like the child itself. Otherwise, it may be difficult for children to keep realizing that the comic is about themselves.

In addition to the comments about the comic and the system, experts gave input on how to design a session to put the comic to use as part of an intervention in the actual school context.

### The Prototype

**Figure 7:**
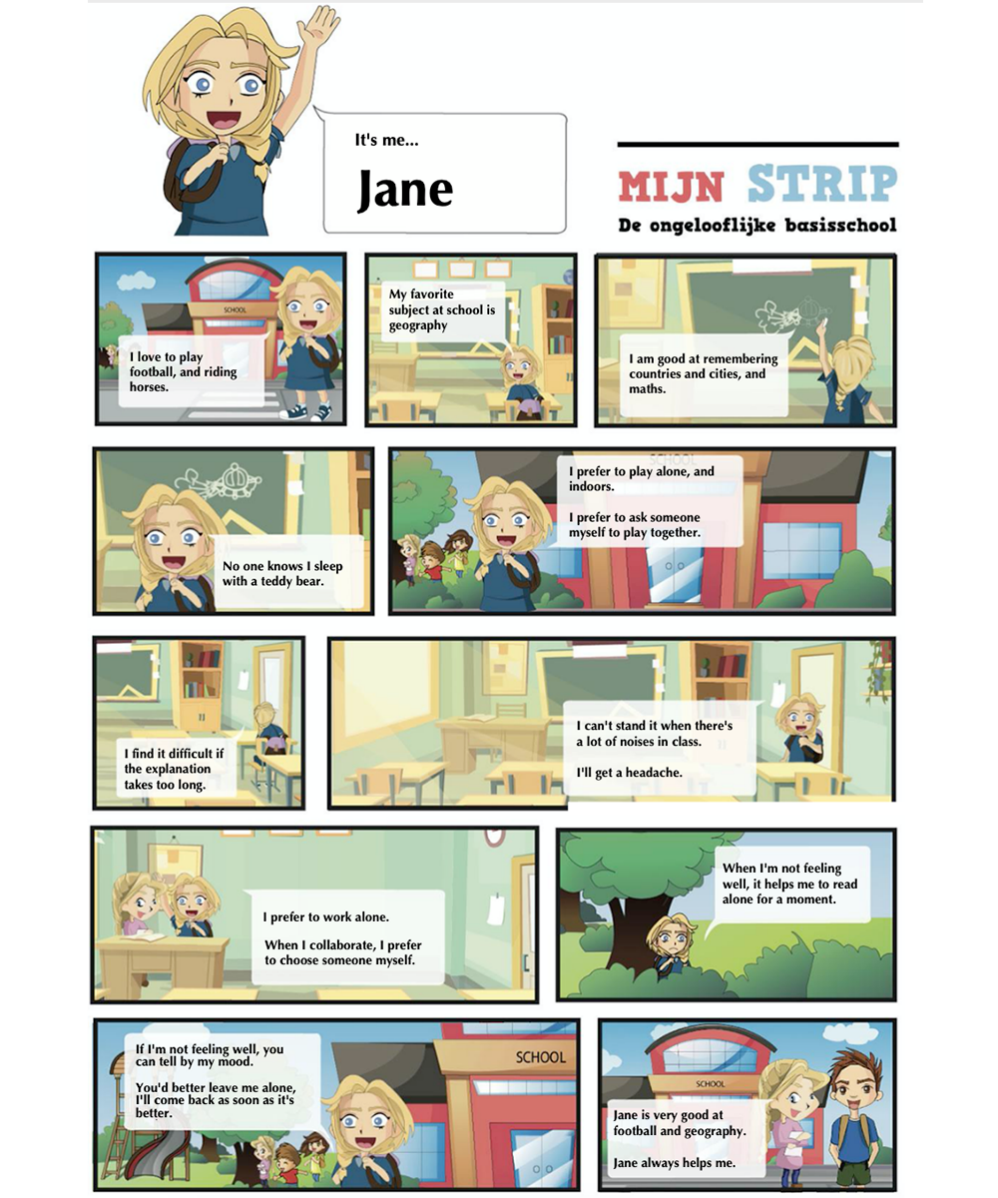
Sample of a generated comic.

For the final prototype, new artwork was created and was produced by a professional illustrator. To keep the system easy to modify, we used Google Forms to create and administer the questionnaire. The advantage of this system is that different people can easily adjust the questions if necessary. By using an add-on (ie, *Form Publisher*), the comics based on the answers from the children are generated on a preset template. These comics are sent as PDF files to the researchers and an email address of choice, usually that of the professional, parent, or involved teacher. Figure 7 shows a sample of a generated comic.

In the artwork and template, all preparations have been made to make the comic strip suitable for personalized characters. However, for this test, we limited the choice between a version with a boy or a girl. If the children did not appreciate the new look and feel during the test, we still had the opportunity to adjust things. Also, in this way, the prototype communicates its flexibility so that different stakeholders still feel invited to provide input. A completed comic can be found in Multimedia Appendix 1.

### Test Session 3

The third test showed that the majority of the children could use the system without help. Children could fill in the questionnaire with relative ease, although some questions required a little more in-depth answers, and children sometimes had to think for a while before they could formulate an answer. Only 2 children with dyslexia experienced more difficulties during the test and needed more help to complete the questionnaire; these children were not diagnosed with ASD. The observations show that children in the first part of the workshop were mainly enthusiastic, as can be seen in Table 1. A single child showed disinterest, and 1 child looked insecure while filling in the questionnaire. A few children became distracted while filling in the questionnaire.

The general tendency during the test session was that when children had finished completing their questionnaires, they started helping the other children. Also, when children got stuck with a specific question, they were often supported by peers or asked each other for help. This behavior occurred naturally without the intervention of the research assistants. The observations also show that in the second phase of the session, the children were mainly helpful, approached each other, or watched each other's screens, as can be seen in Table 2. In only 1 case, a child was making fun of someone else.

Conversations based on the comics were about the personal characteristics of the children. Both the positive and the perceived-as-difficult characteristics of the children were discussed. The general atmosphere during the conversations was safe and thoughtful. In some discussions, children mentioned how they experienced and interpreted their own specific behavior and that of their peers. Children actively gave each other feedback during these conversations. The evaluation (see Table 3) shows that two-thirds of the children knew better how to help peers in the classroom and how to make contact with each other after discussing the comic. More than half of the children indicated afterward that the questionnaire was not very easy to fill in. After asking what the reasons were, this was mainly due to the personal nature of the questions. The personal nature of the items meant that children sometimes had to think carefully. Children were also not very used to questions about this type of personal issue.

The teachers were very positive about the comic and were already thinking about how they could use the tool themselves in their classrooms. One teacher said the following:

It is useful for the beginning of the school year, during the introduction week. Each year, the children are mixed, and all come to a new class. For children with ASD, who are not real talkers, it is useful to get to know each other. The comic is an accessible way to do that.

Another teacher added the following:

I think this tool has added value for everyone. I have a vision of the world of my colleagues and the children in my class. But is that also the vision they have about themselves? I think I know things about you, but is that also the story you would tell about yourself? The tool is a great way to discuss this.

Apart from the fact that teachers reflected on how they would use the tool themselves, during the tests the teachers also learned new things about the children in their classroom. One teacher said the following:

I looked at the comics myself and I was quite surprised by some of them; I got some new insights. You don't just find out particular things when you have a regular conversation...This comic is a bit closer to the children. The comic is about the children themselves instead of about someone else, and that's the beauty of it.

The teachers also see it as an advantage that the teachers themselves do not necessarily initiate the conversation. Through generated comics, discussions arise more naturally. According to the teachers, children are also more honest because the tool makes them feel as though they are really doing this for themselves instead of doing it for school.

The main feedback from the tests focused on the limited possibilities for personalization of the characters in the comic. Both children and teachers found this to be the most significant limitation. Children indicated that they saw personalizing their characters as an element that would increase the fun factor. Teachers appointed a somewhat deeper layer on this topic. One of the teachers formulated it as follows:

Children with autism sometimes focus very much on details, and if the details are not right, then the child will feel less connected to the comic. This also might apply for other children, for example, children with a different cultural background.

**Table 1:** Observations during the first part of the workshop.

Observation time points		Observed behavior (N=47)									
		Enthusiastic, n (%)		Disinterested, n (%)		Angry, n (%)		Insecure, n (%)		Distracted, n (%)	
**1 minute after the test**											
	Observer 1		44 (94)		1 (2)		0 (0)		2 (4)		0 (0)
	Observer 2		44 (94)		1 (2)		0 (0)		2 (4)		0 (0)
**5 minutes after the test**											
	Observer 1		40 (85)		1 (2)		0 (0)		2 (4)		4 (9)
	Observer 2		39 (83)		2 (4)		0 (0)		1 (2)		5 (11)

**Table 2:** Observations during the second part of the workshop.

Observation time points		Social behavior (N=47)			
		Helpful, n (%)	Approaching a peer, n (%)	Watching a peer, n (%)	Making fun of someone, n (%)
**10 minutes after the test**					
	Observer 1	8 (17)	35 (74)	4 (9)	0 (0)
	Observer 2	8 (17)	35 (74)	4 (9)	0 (0)
**15 minutes after the test**					
	Observer 1	12 (26)	13 (28)	11 (23)	1 (2)
	Observer 2	11 (23)	14 (30)	11 (23)	1 (2)

**Table 3:** Evaluation of the questionnaires.

Question	Response to questions^a^ (N=47)			
	Yes, n (%)	Neutral, n (%)	No, n (%)	Blank, n (%)
1. I thought it was a fun assignment to do.	39 (83)	6 (13)	2 (4)	0 (0)
2. I like the way it looks.	33 (70)	12 (26)	2 (4)	0 (0)
3. The questions are easy to answer.	20 (43)	23 (49)	4 (9)	0 (0)
4. I now know better what the others are good at and what the others are less good at.	28 (60)	16 (34)	3 (6)	0 (0)
5. I know better now how I can help others.	30 (64)	12 (26)	4 (9)	1 (2)
6. I know better now how to make contact.	28 (60)	14 (30)	5 (11)	0 (0)
7. Discussing the comic was easy for me.	28 (60)	14 (30)	4 (9)	1 (2)
8. I'd like to do this assignment again.	29 (62)	12 (26)	6 (13)	0 (0)

^a^Questions were answered using smileys, as seen in Figure 6; in that figure, the green smiley on the left indicates “yes,” the yellow smiley in the middle indicates “neutral,” and the red smiley on the right indicates “no.”

## Discussion

### Principal Findings

Children with ASD regularly face challenges in social situations and are more likely to be excluded by peers. Many interventions for children with ASD are focused on improving social skills. Although many interventions demonstrate that targeted social skills can be improved in clinical settings, developed social skills do not necessarily get applied in children's daily lives at school [11,18]. One of the possible reasons for this is that classmates continue to show negative bias toward children with ASD, even when children do improve social skills [19]. Children with ASD have a higher risk of being victims of peer harassment and peer exclusion [12-15]. Although social skills training can contribute to social functioning and, therefore, more social acceptance, children with ASD do not necessarily experience this link directly [20].

This study focused on developing a tool to contribute to more mutual understanding among children with and without ASD at school. In this study, we designed the tool *It's me*, a medium that creates a personal comic about someone's talents and perceived difficulties. Comics as a medium hold the promise to trigger different affective processes of, and responses by, readers [33,34], which makes them suitable to tell complex and emotionally rich stories [35]. Through *It's me*, children with and without ASD can tell their stories about who they are in a light and accessible way. Although the personal questions are sometimes tricky to answer for the children, the comic has shown its potential to initiate personal conversations between children in which they learn new things from each other and gain more understanding for each other. After the tests, teachers indicated that they had gained new insights from the information in the comics and that they found *It's me* useful for increasing social acceptance in their classroom.

Based on concepts of peer support [61], psychoeducation [62], and horizontal interaction, *It's me* is based on different underlying concepts that have been translated into design. The tests seem to reflect these underlying concepts, at least for the period during and just after the use of *It's me*. The main idea of *It's me* assumes that greater understanding between children leads to less peer harassment and increased inclusion of children who are different in a certain way. Teachers are convinced that *It's me* can be of added value in their classroom and that *It's me* can contribute to an improved group dynamic. *It's me* tries to create a safe context at school in which learned social skills should ideally be applied. *It's me* does not aim to replace existing social skills training but attempts to be complementary by intervening from a different perspective and in a different context.

Equivalence between the participants is an essential element in the application of *It's me*. The developed tool consciously focuses on both talents as well as on topics where children perceive more difficulty. Based on a horizontal interaction principle, *It's me* tries to contribute to the development of better relationships between children. Teachers indicate that they see the potential to use the tool at the beginning of a school year, to provide a good start in social acceptance in their class. They also mention a practical reason: by using the tool at the beginning of the school year, they can look back on it from time to time and reflect with the children if necessary.

The tool seems promising based on these first results but requires future work on two themes. The first theme is to finish the development phase of the tool and add a few functionalities. This development includes more features to personalize the comic, regarding both the characters and the background pictures. The second theme is to design a series of lessons. These lessons can be a manual for facilitators that will help guide them in the usage of the tool and to give them directions on how to lead the conversations based on the comics. For a facilitator, it is essential to guarantee a safe atmosphere during the conversations. However, at the same time, facilitators should create depth within the discussions if this does not occur naturally. By using the tool in a targeted way, it is possible to investigate the sustainable effect among students in classes over a more extended period, which is an interesting topic for future research.

During the design process, we tested several prototypes with different stakeholders. Many insights were gained from the reactions of the stakeholders to the prototypes. During the test sessions, it was established that various stakeholders, including high-functioning children with ASD and their peers, professionals, and teachers, exposed processes of perspective taking and making. These processes can be linked to the reflection learning mechanism that boundary objects can enact [40]. The first signs of a common problem space, necessary for transformation, manifested itself at the end of the design research process, during the conversations based on the comics and during the evaluation with teachers.

Through prototypes, the intervening effects of the tool within a social system became apparent in the early stages of the design process. As a result, various design constraints have been localized. The importance of a safe atmosphere during the conversations is one of those constraints. When localized, constraints can be addressed within the rest of the design process. Through testing with prototypes, it also became evident whether the prototypes could function as boundary objects and, thus, whether the tool would be able to adapt to the local needs and constraints of different parties. The focus on this feature of boundary objects was essential in finding the right design. In the specific case of this study, you can see that the developed tool was experienced as something useful for professionals, teachers, and children, albeit from a different perspective. The professionals see the potential of the tool to create a more constructive context at school for the application of skills that children learn in social skills training. Children with ASD see a fun tool that helps them to initiate a conversation with peers. Finally, teachers see added value in the tool in making difficult personal themes discussable in the classroom.

The design research approach we used in this study, which aimed to design an intervention that successfully functions as a boundary object, might be beneficial in the acceptance and adoption of the intervention. Because boundary objects are addressing different local needs, everyone identifies with a boundary object in a certain way. In the design process of *It's me*, the learning mechanisms of identification, coordination, and reflection passed in sequence among the different stakeholders. The applied research and design strategy helped us to continuously monitor whether the developed tool had added value for the various stakeholders. This strategy can be interesting for other designers and researchers who are dealing with a case involving multiple stakeholder groups or social-cultural systems that have different objectives. Boundary objects can bridge the gap between various stakeholders by representing different objectives from different interpretations without prioritizing an objective of a specific social-cultural system.

### Conclusions

Children with ASD regularly face challenges in social interactions, especially at school. In this study, a serious media digital tool, *It's me*, was designed to facilitate and enact horizontal communication between high-functioning children with ASD and their peers at school. Based on concepts of peer support and psychoeducation, *It's me* aims to initiate in-depth conversations between peers, which should lead to more mutual understanding and better relationships. The first test sessions showed that the tool was easy to use for the target group, and the tool seemed to have the potential to initiate personal conversations among children. *It's me* was designed as a boundary object that aims to connect the objectives of different stakeholders. Due to this focus during the design process, we gathered a lot of insights about the potential of the tool along the way. The applied design research approach might be of added value in the acceptance and adoption of the intervention, because children, professionals, and teachers saw added value in the tool, each from their own perspectives.

## Appendix

Multimedia Appendix 1 A completed comic (in Dutch).

## Abbreviations

ASD: autism spectrum disorder
