# Supplementary material for: Design of a Digital Comic Creator (It’s Me) to Facilitate Social Skills Training for Children With Autism Spectrum Disorder: Design Research Approach
Source: JMIR Ment Health. 2020 Jul 10;7(7):e17260. doi: 10.2196/17260 (PMC7382019; doi:10.2196/17260)

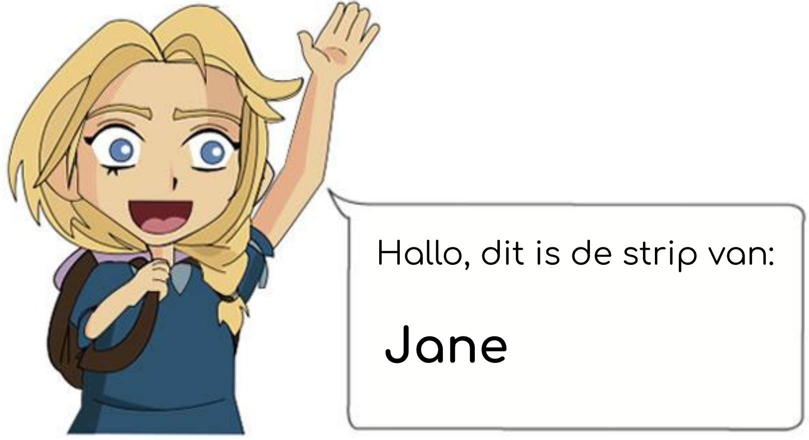

MIJN STRIP

De ongelooflijke basisschool

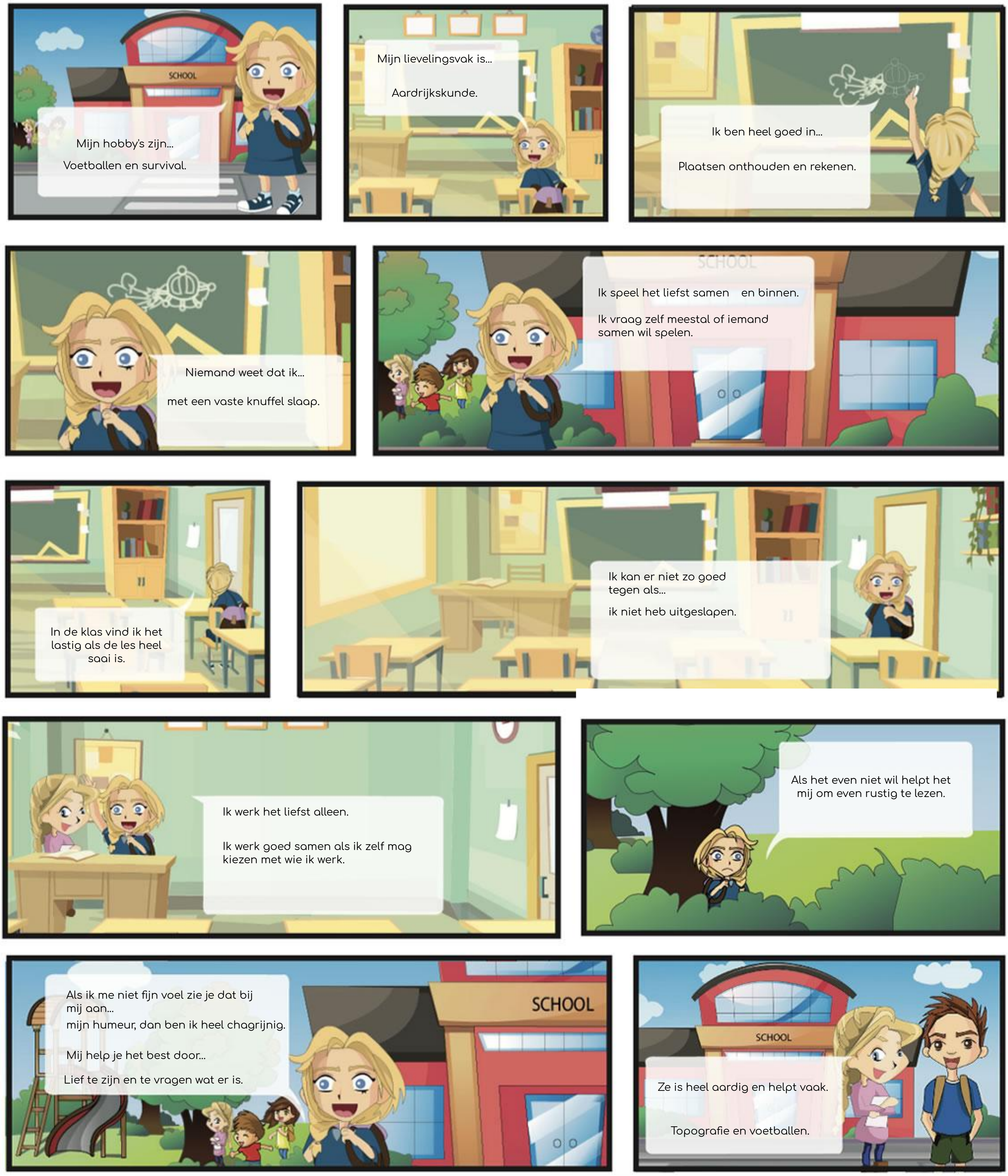

Supplement: Multimedia Appendix 1 [file mental_v7i7e17260_app1.pdf]
